# Survival in Huntington’s disease and other young‐onset dementias

**DOI:** 10.1002/gps.5913

**Published:** 2023-04-16

**Authors:** Samantha M. Loi, Paraskevi Tsoukra, Emily Sun, Zhibin Chen, Pierre Wibawa, Maria di Biase, Sarah Farrand, Dhamidhu Eratne, Wendy Kelso, Andrew Evans, Mark Walterfang, Dennis Velakoulis

**Affiliations:** ^1^ Neuropsychiatry NorthWestern Mental Health, Melbourne Health Royal Melbourne Hospital Parkville Victoria Australia; ^2^ Department of Psychiatry The University of Melbourne Parkville Victoria Australia; ^3^ Department of Neurology Evaggelismos Hospital Athens Greece; ^4^ School of Public Health and Preventive Medicine Monash University Clayton Victoria Australia; ^5^ Florey Institute of Neuroscience and Mental Health Parkville Victoria Australia; ^6^ Department of Medicine Royal Melbourne Hospital Parkville Victoria Australia

**Keywords:** dementia, early‐onset dementia, epidemiology, Huntington's disease, mortality, prognosis, risk factors, survival, young‐onset dementia

## Abstract

**Objectives:**

To compare survival and risk factors associated with mortality in common young‐onset dementias (YOD) including Huntington's disease.

**Methods:**

This retrospective cohort study included inpatients from an Australian specialist neuropsychiatry service, over 20 years. Dementia diagnoses were based on consensus criteria and Huntington's disease (HD) was confirmed genetically. Mortality and cause of death were determined using linkage to the Australian Institute of Health and Welfare National Death Index.

**Results:**

There were 386 individuals with YOD included. The dementia types included frontotemporal dementia (FTD) (24.5%), HD (21.2%) and Alzheimer's disease (AD) (20.5%). 63% (*n* = 243) individuals had died. The longest median survival was for those who had HD, 18.8 years from symptom onset and with a reduced mortality risk compared to AD and FTD (hazard ratio 0.5). Overall, people with YOD had significantly increased mortality, of 5–8 times, compared to the general population. Females with a YOD had higher standardised mortality ratio compared to males (9.3 vs. 4.9) overall. The most frequent cause of death in those with HD was reported as HD, with other causes of death in the other YOD‐subtypes related to dementia and mental/behavioural disorders.

**Discussion:**

This is the first Australian study to investigate survival and risk factors of mortality in people with YOD. YOD has a significant risk of death compared to the general population. Our findings provide useful clinical information for people affected by YOD as well as future planning and service provision.

## Key points

- Dementia is a leading cause of death in Australia and the leading cause of death in females. There is limited mortality information pertaining to younger people with dementia.
- We investigated mortality and risk factors in young‐onset dementia, including Huntington's disease. Huntington's disease had the longest survival ‐ 18.8 years, with Alzheimer's disease, frontotemporal and vascular dementias having 11 and 13 years median survival. Young‐onset dementia conferred between 5 and 8 times increased risk of death compared to the general population.
- Young‐onset dementia is a fatal disease with shortened lifespan. Early identification and timely age‐appropriate services are necessary for these individuals.

## 1 INTRODUCTION

Young‐onset dementia (YOD) refers to a dementia where symptom onset occurs at less than 65 years of age.^1^ A recent Delphi study reported high consensus that the most common causes of dementia in younger people include Alzheimer's disease (AD), frontotemporal dementia (FTD), vascular dementia (VaD) and Huntington's disease (HD).^2^ While AD, FTD and VaD have both sporadic and genetic causes, HD is relatively rare in comparison, with a reported prevalence of 5.7 per 100,000,^3^ and is an autosomal dominant neurodegenerative condition caused by a trinucleotide CAG repeat expansion on chromosome 4, causing abnormal accumulation of the huntingtin protein (*HTT*).^4^ The characteristic triad of symptoms are neurological, cognitive and psychiatric, with cognitive and psychiatric symptoms preceding chorea by up to 15 years.^5^ Individuals with 36–39 CAG repeats will be at increased risk of developing symptoms related to HD and those with ≥40 CAG repeats will have 100% complete penetrance and develop HD.^5^ A higher number of CAG repeats is associated with development of symptoms at a younger age, faster progression and earlier age of death.^6^

Dementia is a leading cause of death in older people and^7^ investigating survival in dementia has important implications for societal costs and disease burden, and in YOD, is essential for future planning and disease progression due to its onset in midlife.^8^, ^9^ There has not been any previous work comparing survival in these major types of YOD in Australia. Survival in non‐genetic causes of YOD has been reported as 22 years for VaD, 16.4 years for FTD and 15.6 years for AD, from symptom onset.^10^ Shorter survival duration was reported in a larger Dutch cohort, with 7 years survival from age at diagnosis for FTD and AD.^11^ Survival in HD has ranged from 14.5^12^ to 35 years from symptom onset.^13^ Our previous work in this area found that the mean survival time in YOD overall was 12.7 years, double that of older‐onset dementia (6.3 years)^14^ but excluded individuals with HD. In this study, the main objective was to determine survival of individuals with HD compared with other YODs, in a well‐characterised cohort seen over a 20‐year period. Secondary objectives were to identify factors associated with survival, to compare mortality rates to Australian population norms, and to investigate causes of death.

## 2 MATERIALS AND METHODS

### 2.1 Participants and study design

This retrospective study was performed at Neuropsychiatry, Royal Melbourne Hospital, Australia. ‘Neuropsychiatry’ is a tertiary specialist centre providing assessment, diagnosis and follow‐up of people with a range of neuropsychiatric conditions, including YOD. Neuropsychiatry also has a HD‐specific service including a predictive testing clinic for those at risk of HD.^15^ Inpatients are admitted for multidisciplinary assessment (neuropsychiatry, neurology, allied health and nursing) and investigations (neuroimaging, lumbar puncture and blood tests). The detailed methods have been previously described.^14^

We reviewed all inpatient summaries for the period 1992–2014 inclusive and identified patients diagnosed with any dementia diagnosis during this period. Individuals were included if they had symptoms determined to be related their dementia at less than 65 years old. Data collection included:

- Demographics;
- Dementia diagnoses: Alzheimer's disease (AD),^16^ frontotemporal dementia, FTD (including behavioural‐^17^ and language‐variants,^18^ FTD motor neuron disease, FTD‐MND, corticobasal syndrome [CBS] and progressive supranuclear palsy [PSP]), VaD (including mixed AD and VaD) and HD. All other dementia diagnoses were categorised as ‘Other Dementia’, including dementia with no specified aetiology. Dementia diagnoses were reported as the dementia type reported at the most recent follow‐up appointment;
- Earliest presenting symptoms attributed to the dementia was defined as age of onset and were categorised as cognitive, psychiatric/behavioural or neurological;
- Comorbidities such as alcohol use, psychiatric history, family history of dementia and psychiatric disorders and cardiovascular risk factors (CVRFs) (such as hypertension and smoking);
- Cognition, utilising the Neuropsychiatry Unit cognitive assessment tool (NUCOG).^19^ Five cognitive domains of attention, memory, visuospatial, executive and language function are assessed with total score of 100. Higher scores indicate better cognition.
- Mortality information was identified using the Australian Institute of Health and Welfare (AIHW) via the Australian National Death Index (NDI). The NDI contains information relating to all deaths after 1980. There were two linkage dates (30/9/2019 and 19/2/2020) with 100% of all names linked.

### 2.2 Statistical analysis

*Event time* was defined as the time of death. The Kaplan‐Meier method was used to estimate the median survival from age of onset. Survival curves of dementia types were compared using log rank test. To examine independent predictors of survival (dementia subtype, comorbidities, presenting symptoms), a Cox proportional hazards model was used. Standardised mortality ratios (SMRs) for the study population were estimated from published age‐, sex‐ and year‐specific mortality rates available from the Australian Bureau of Statistics (ABS). Significance level was set at *p* < 0.05 with Holm‐Bonferroni correction for multiple comparisons. Statistical analyses were performed using Stata 14 (StataCorp) and Statistical Package for the Social Sciences (version 26, IBM Corporation).

This study was approved by the Melbourne Health (2016.038) and the AIHW human research ethics committees (EO2017/5/398).

## 3 RESULTS

### 3.1 The whole cohort

Of 568 inpatients with a dementia diagnosis, there were 42 patients whose age of onset was not clear, *n* = 25 had primary psychiatric disorders with cognitive impairment and *n* = 115 had older‐onset dementia, who were excluded. The final cohort included 386 inpatients. 194 (50.3%) of the 386 participants were male and the average age at symptom onset was 51.2 years (SD = 10.2, range 13, 67). The mean age of admission was 54.4 years (SD = 10.3, range 15, 87). The majority of patients were Caucasian (*n* = 360, 93.3%), living at home prior to admission (*n* = 277, 71.8%) and were secondary‐school‐educated (*n* = 223, 68.0%). The mean NUCOG score was 66.0 (SD = 16.8, range 16, 98.5), indicating moderate cognitive impairment. 45.7% (*n* = 170) had initial presenting symptoms which were cognitive in nature and *n* = 155 (40.2%) had psychiatric symptoms. 61.9% inpatients had a previous psychiatric history (*n* = 237).

There were 82 inpatients with HD (21.2%) and their mean CAG repeat was 45.4. *N* = 94 (24.4%) had FTD, *n* = 86 (22.3%) had Other Dementia, *n* = 79 (20.5%) had AD and *n* = 45 (11.7%) had VaD. Individuals with Other Dementia included Parkinson's disease dementia (*n* = 14), Dementia with Lewy bodies (*n* = 14), acquired brain injury (*n* = 6) and alcohol‐related dementia (*n* = 17) (Table 1).

**TABLE 1:** Demographics of the *n* = 386 inpatients.

	Number (%)	Missing data (%)
Males	194 (50.3)	
Ethnicity		1 (0.8)
Caucasian	360 (93.3)	
Asian	11 (2.9)	
Southern and central Asia	3 (0.8)	
North Africa/Middle East	9 (2.3)	
Location		3 (0.8)
Metropolitan	248 (64.8)	
Rural/regional	120 (31.3)	
Interstate	15 (3.9)	
Location prior to admission		
Home	277 (71.8)	
Hospital	38 (9.8)	
Psychiatric inpatient unit	40 (10.4)	
Residential facility	31 (8.0)	
Education		58 (15.0)
Primary	27 (7.0)	
Secondary	223 (68.0)	
Tertiary	77 (19.9)	
Other (special school)	1.0 (0.3)	

### 3.2 Dementia subtypes

Table 2 shows information relevant to dementia subtypes. Inpatients with HD had the youngest age of onset, 43.7 years (SD = 10.7), significantly lower compared to the other 306 inpatients with YOD (*M* 43.5 cf *M* 52.9, *t*(365) = 7.270, *p* < 0.001). Compared to the other YODs, a higher proportion of inpatients with HD reported a current or previous heavy alcohol history (35.4% vs. 25%, *χ* ^2^(2) = 15.437, *p* < 0.001) and a lower proportion of them had CVRFs present (32.9% vs. 58.2%, *χ* ^2^(1) = 16.585, *p* < 0.001). Not unexpectedly, more individuals with HD (73%) had a positive family history, compared to the other dementia subtypes, *χ* ^2^(8) = 23.8, *p* = 0.002.

**TABLE 2:** Clinical characteristics of the young‐onset dementia subtypes, *n* = 386.

	Alzheimer's disease	Frontotemporal dementia	Huntington's disease	Other dementia	Vascular dementia	
*N* (% of total)	79 (20.5)	94 (24.5)	82 (21.2)	86 (22.3)	45 (11.7)	
Mean age of onset (SD)	54.7 (7.4)	51.3 (8.6)	43 (10.5)	51.3 (12.2)	56.7 (7.2)	F(4,242) = 5.671, *p* < 0.001*
Mean admission age (SD)	58.1 (7.2)	54.1 (8.7)	47.9 (12.2)	54.6 (10.9)	60.4 (5.7)	F(4,385) = 7.408, *p* < 0.001*
*N* males (% of dementia subtype)	31 (39.2)	46 (47.9)	32 (39.0)	61 (70.9)	24 (53.3)	*χ* ^2^(4) = 24.76, *p* < 0.001*
	Adj −2.9		Adj −2.2	Adj 3.9		Other > HD > AD
CVRF—yes (%)	35 (44.3)	48 (51.1)	27 (32.9)	51 (59.3)	43 (95.6)	*χ* ^2^(4) = 49.87, *p* < 0.001*
			Adj −4.1		ADj 6.1	VaD > HD
Number of CVRF (SD)	0.6 (0.8)	0.9 (0.9)	0.5 (0.74)	0.9 (0.9)	2.1 (1.1)	F(4,385) = 21.14, *p* < 0.001*
Psychiatric history—yes (%)	46 (58)	54 (56.4)	48 (59.5)	56 (65.1)	35 (77.8)	*χ* ^2^(4) = 7.05, *p* = 0.134
Family history—yes (%)	40 (51.3)	48 (51.6)	59 (73.8)	41 (47.1)	19 (42.2)	*χ* ^2^(8) = 23.80, *p* = 0.002
Alcohol use—yes (%)	19 (24.1)	22 (23.4)	29 (35.4)	24 (27.9)	11 (24.0)	*χ* ^2^(8) = 15.97, *p* = 0.043
Presenting symptoms psychiatric/behavioural (%)	19 (24.1)	57 (60.6)	30 (43.5)	30 (35.3)	19 (42.2)	*χ* ^2^(8) = 78.85, *p* < 0.001*
	Adj −3.6	Adj 4.7				FTD > AD
Neurological/motor (%)	4 (5.1)	6 (6.4)	25 (36.2)	8 (9.4)	4 (8.9)	HD > FTD > AD
	Adj −2.3	Adj −2.1	Adj 6.5			
Cognitive (%)	56 (70.9)	31 (33.0)	14 (20.3)	47 (55.3)	22 (48.9)	AD > HD > FTD
	Adj 5.0	Adj −2.9	Adj −4.7			
Mean NUCOG total (SD)	60.5 (15.7)	64.0 (20.9)	69.9 (14.3)	69.8 (14.5)	64.8 (17.2)	F(3,181) = 2.50, *p* = 0.044
*N* dead (% total)	56 (23)	62 (20.4)	44 (18.1)	50 (20.6)	31 (12.8)	
*N* dead (% of dementia subtype)	56 (70.9)	62 (66.0)	44 (53.7)	50 (58.1)	31 (68.9)	*χ* ^2^(4) = 7.07, *p* = 0.132
Mean age at death (SD)	65.7 (9.4)	60.8 (11.1)	57.1 (13.6)	62.2 (11.2)	67.3 (5.6)	F(4,242) = 6.11, *p* < 0.001*
Median survival years, (95% CI)	11.3 (9.8, 12.9)	10.6 (8.9, 13.2)	18.8 (12.8, 21.5)	14.5 (12.8, 18.2)	12.3 (9.4, 16.1)	*χ* ^2^(4) = 11.04, *p* = 0.026

*Note*: Alzheimer's disease includes *n* = 6 individuals with posterior cortical atrophy. Frontotemporal dementia (FTD) includes: *n* = 4 FTD‐motor neuron disease, *n* = 6 progressive non‐fluent aphasia, *n* = 5 semantic dementia, *n* = 74 behavioural‐variant FTD, *n* = 3 corticobasal syndrome *n* = 2 progressive supranuclear palsy. Other Dementia includes: *n* = 26 unknown/other, *n* = 14 dementia with Lewy bodies, *n* = 14 Parkinson's disease dementia, *n* = 17 alcohol‐related dementia, *n* = 6 Niemann‐Pick type C, *n* = 6 acquired brain injury, *n* = 2 multiple system atrophy; *n* = 2 dementia related to multiple sclerosis; *n* = 1 related to MELAS; *n* = 1 neurosyphillis; *n* = 1 meningitis.

Abbreviations: CI, confidence intervals; CVRF, cardiovascular risk factors; NUCOG, Neuropsychiatry Unit Cognitive Assessment; SD, standard deviation; YOD, young‐onset dementia.

Patients with VaD were older at symptom onset (*M* age 56.7 years) compared to patients with HD (*M* age 43.7 years, *p* < 0.001) FTD and Other Dementias (*M* age 51.3 years, *p* = 0.001) and more patients with VaD had the presence of CVRF, *χ* ^2^(4) = 49.9, *p* < 0.001 and a higher number of CVRF (*p* < 0.001), compared to the other dementia subtypes. There was no difference in psychiatric history between the dementia subtypes.

### 3.3 Mortality

**FIGURE 1:**
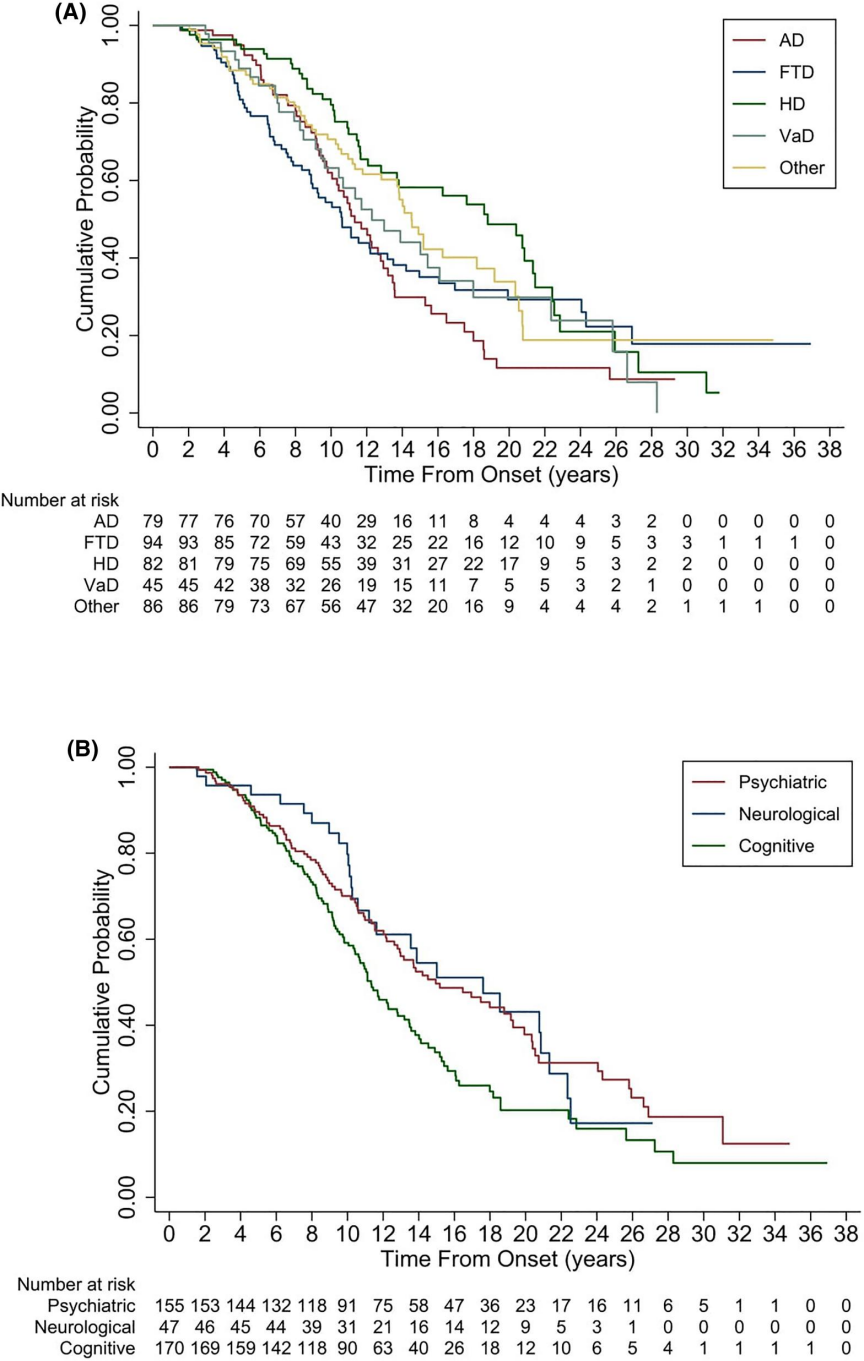
(A) Kaplan Meier curve for survival for young‐onset dementia. (B) Kaplan Meier survival curves for individuals with young‐onset dementia, depending on the initial presenting symptoms.

*N* = 243 (63%) of the inpatients had died. The mean age at death was 62.4 years (SD = 11.2, range 18, 89.5). There was no difference in the proportions of people who had died in each dementia group *χ* ^2^(4) = 7.07, *p* = 0.132. Those with HD had the youngest age at death (*M* age 57.1 years), compared to AD (*M* age 65.8 years), *p* = 0.001 and those with VaD (*M* 67.3 years), *p* = 0.01. Median survival for HD was 18.8 years (95% CI 12.1, 21.5) and for the other dementia subtypes were: AD 11.3 years (95% CI 9.8, 12.9), FTD 10.6 years (95% CI 8.9, 13.2), Other Dementias 14.5 years (95% CI 12.8, 18.2) and VaD 12.3 years (95% CI 9.4, 16.1) (Figure 1A). Individuals with HD had the longest survival, *p* = 0.02.

Median survival was similar for males (13.7 years, 95% CI 12.0, 15.1) compared to females (12.1 years, 95% CI 10.6, 15.2), *p* = 0.151, with no differences in the proportions of males (62.9%) or females (63.0%) who died, *χ* ^2^(1) = 0.001, *p* = 0.978. Figure 1B shows the Kaplan Meier curve for survival depending on the type of symptom presentation for the dementia, with cognitive presentation being the shortest median duration, 11.3 years (95% CI 10.33, 12.82), *p* = 0.006, compared to psychiatric presenting symptoms (median 14.9 years, 95% CI 12.6, 19.3) and neurological presenting symptoms (median 17.6 years, 95% CI 1.2, 21.3).

The mortality risk was greatest in people with FTD (SMR = 8.0, 95% CI 6.2, 10.3), followed by people with HD (SMR = 7.3, 95% CI 5.4, 9.8), VaD (SMR 5.9, 95% CI 4.1, 8.3), AD (SMR = 6.0, 95% CI 4.6, 7.8) and Other dementia (SMR = 5.3, 95% CI 4.0, 7.0). For all YOD diagnoses, females had increased SMR compared to population norms (Other Dementias SMR = 19.6; FTD SMR = 11.9; HD SMR = 8.5, VaD SMR 7.7; and AD SMR = 6.8) and compared to males, and almost double that of males for FTD and Other Dementias (Table 3). Having a YOD had an overall risk of death 6.4 times greater than the general population (95% CI 5.7, 7.3). Being female and having a YOD conferred a 9.3 times risk of death compared to the general population (95% CI 7.8, 11.1) and being male with a YOD had a SMR 4.9 (95% CI 4.1, 5.8). Compared to the general population, from ages 50–59, the risk of death was 8.1 times (95% CI 6.2, 10.7) and ages 60–69 was 6.5 times (95% CI 5.3, 8.0).

**TABLE 3:** SMRs for young‐onset dementia subtypes and sex.

		Observed death	Expected	SMR	95% confidence interval
Dementia	Sex				
Alzheimer's disease	All	56	9.29	6.03	4.64, 7.83
	Female	32	4.70	6.80	4.81, 9.62
	Male	24	4.59	5.23	3.51, 7.81
Frontotemporal dementia	All	62	7.76	7.99	6.23, 10.25
	Female	34	2.86	11.91	8.51, 16.67
	Males	28	4.90	5.71	3.94, 8.27
Huntington's disease	All	44	6.01	7.32	5.44, 9.83
	Female	25	2.95	8.49	5.73, 12.56
	Male	19	3.07	6.19	3.91, 9.71
Other dementia	All	50	9.51	5.26	3.98, 6.93
	Female	18	0.92	19.62	12.36, 31.14
	Male	32	8.60	3.72	2.63, 5.26
Vascular dementia	All	31	5.29	5.86	4.12, 8.33
	Female	13	1.68	7.74	4.94, 13.33
	Mare	18	3.61	4.99	3.14, 7.91
Age category	30–39	11	0.25	44.58	24.69, 80.49
	40–49	22	1.13	19.45	12.81, 29.54
	50–59	50	6.16	8.12	6.16, 10.72
	60–69	99	15.12	6.54	5.38, 7.97
	70–79	53	10.65	4.98	3.80, 6.52
	80–89	7	4.38	1.60	0.76, 3.50

Abbreviation: SMR, standardised mortality rates.

### 3.4 Predictors of mortality in YOD

The Cox regression model (Table 4) was significant in finding an association between mortality risk (*χ* ^2^(8) = 23.89, *p* = 0.0024). There were two significant predictors of association. Having HD, compared to AD and FTD, was associated with a lower mortality risk, with HR = 0.55 (*p* = 0.009) and HR = 0.52 (*p* = 0.004), respectively. Patients who initially presented with symptoms that were cognitive in nature as part of their dementia, had increased hazard ratio (HR 1.54, 95% CI 1.14, 2.07, *p* = 0.005) compared to those presenting initially with psychiatric symptoms. Neither CVRFs nor alcohol were significantly associated as predictors of mortality.

**TABLE 4:** Cox proportional regression/hazard ratios for predictors of association for mortality in young‐onset dementia.

	Hazard ratio	*z*	*p* Value	95% confidence interval
Dementia type^a^				
Frontotemporal dementia	1.05	0.27	0.786	0.72, 1.55
Huntington's disease	0.55	−2.62	0.009	0.35, 0.86
Other dementia	0.67	−2.03	0.042	0.45, 0.99
Vascular dementia	0.86	−0.64	0.522	0.58, 1.49
Alcohol—Yes	1.02	0.15	0.883	0.76, 1.38
CVRF—Yes	1.12	0.78	0.434	0.84, 1.48
Presenting symptom^b^				
Cognitive	1.54	2.84	0.005	1.14. 2.07
Neurological	1.18	0.72	0.471	0.75, 1.87

Abbreviation: CVRF, cardiovascular risk factors.

^a^ Reference group, Alzheimer's disease.

^b^ Reference group, psychiatric presenting symptoms.

### 3.5 Cause of death

**FIGURE 2:**
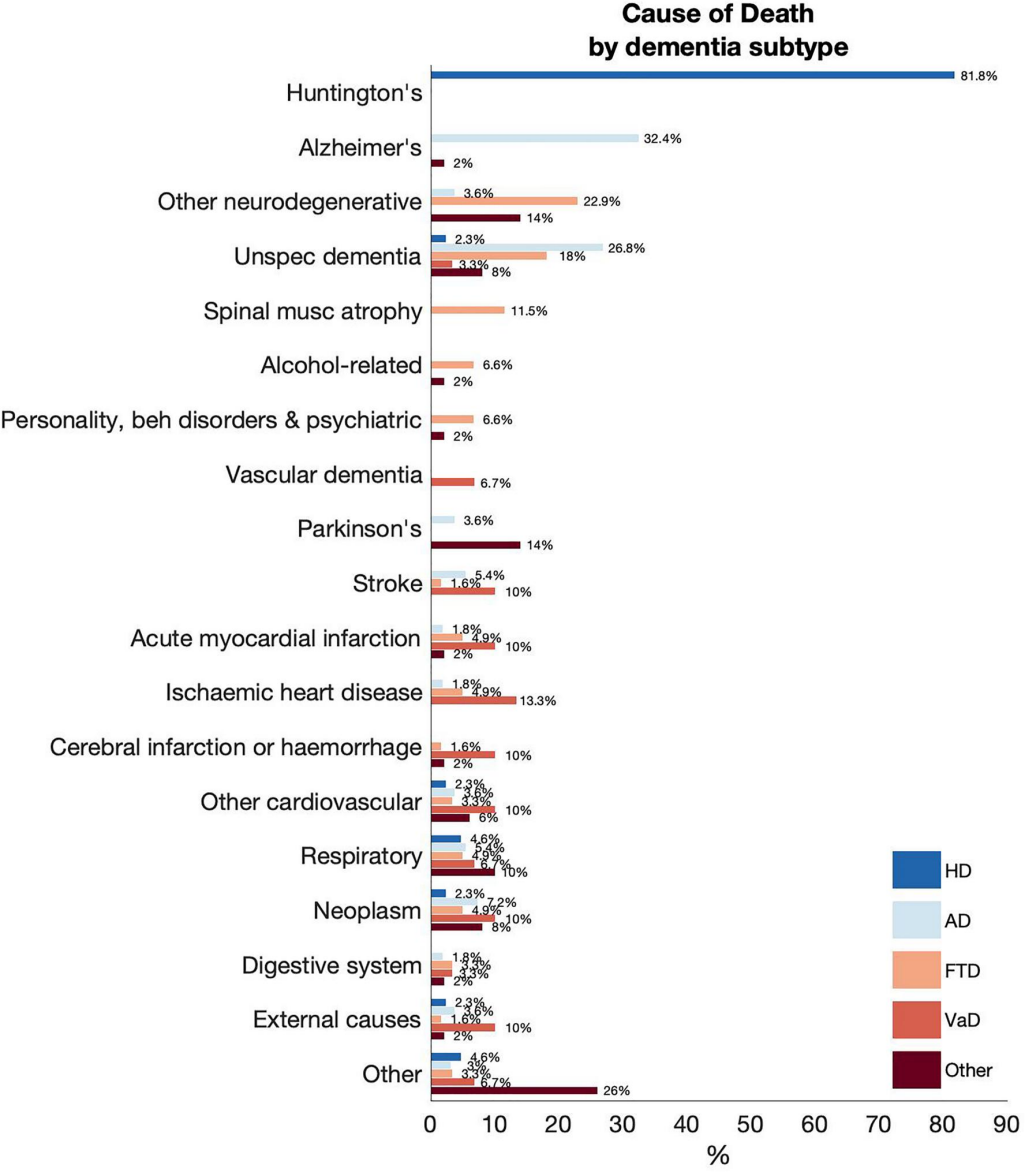
Underlying causes of death for young‐onset dementia subtypes.

The underlying/primary cause of death (Figure 2) was obtained for almost all the individuals (*n* = 2 unknown). Individuals with HD had their primary cause of death reported as ‘Huntington's disease’ (*n* = 36, 81.8%), and one each as ‘external causes’, ‘neoplasm’ and ‘unknown’. Overall, the most frequent underlying cause of death in 97 individuals (39.9%) was ‘Diseases of the nervous system’ (*n* = 23/56 AD, 41.1%; *n* = 21/62 FTD, 33.9%; *n* = 17/50; *n* = 36/44 HD, 81.8%; Other Dementia, 34%) and ‘Mental and behavioural problems’ (total *n* = 45/243, 18.5%; *n* = 16/56 AD, 28.6%; *n* = 19/62 FTD, n = 1/44 HD, 2.3%; 33.9%; *n* = 6/50 Other dementia, 12%). ‘Diseases of the circulatory system’ was the most frequent underlying cause of death for individuals with VaD (*n* = 14/31, 45.2%). ‘External’ or ‘unknown’ causes of death were attributed to nine individuals.

## 4 DISCUSSION

This is the first study to examine mortality in the commonest causes of YOD in Australia, using the NDI to obtain complete, reliable information and minimise attrition.^11^, ^20^ We found that HD had longest survival (18.8 years) compared to the other dementia subtypes. All YOD subtypes were associated with increased mortality (5–8 times greater than the population norms). Our study identified three additional findings. First, having HD compared to AD and FTD conferred lower mortality risk. Second, presenting with cognitive symptoms as part of a dementia was a risk factor for mortality, compared to presenting with psychiatric symptoms, which we had previously found.^14^ Finally, females with YOD had higher SMR compared to males. Our findings emphasise the need for early identification of dementia and provide clinicians with evidence‐based information for the important questions frequently asked by people with YOD and their families.

Our study reported that those with HD had the longest survival, which has been reported elsewhere.^12^, ^13^ It remains unclear why people with HD have such a long survival, particularly in comparison to the other YODs. The CAG repeat length has been inconsistently associated with survival duration^6^, ^12^ with alternative genetic and environmental factors affecting survival in this group.^6^, ^21^ Different atypical presentations in HD might influence survival. Older individuals with HD have mild or subtle cognitive manifestations which do not come to attention until their motor symptoms become more obvious.^21^ Younger people who are gene‐positive for HD who present with psychosis during early adulthood have poorer outcomes in terms of cognition, behaviour and survival.^22^ These individuals appear to have less chorea, even after adjusting for antipsychotic use, suggesting a distinct clinical course.^22^

HD was found to have half the risk of death and longer survival duration compared to AD and FTD. 30%–50% of individuals with FTD have a genetic cause, with the most common autosomal dominant form caused by the chromosome 9 open reading frame 72 (*C9orf72*) mutation.^23^ Comparing genetic and sporadic forms of FTD and survival suggests that having a pathogenic mutation confers increased mortality^24^ but there is variation within these mutations. The microtubule‐associated‐protein tau (*MAPT*) mutation appears to be associated with shorter survival compared to the progranulin and *C9orf72* mutation.^25^ The *C9orf72* mutation is a cause of FTD, MND and FTD‐MND^23^ and while there is consistency that individuals with FTD‐MND phenotype have a short survival, 2–3 years,^26^ there are increasing reports that there are others who have a range of presentations^27^ and outcomes, with some people living for a very long time.^28^ For genetic forms of AD, there is variation in symptom onset, with individuals who had a presenilin 1 (*PSEN1*) mutation having onset on average 7.1 years earlier than those with amyloid precursor protein (*APP*) mutation. However, their median survival was similar for both pathogenic mutations, 11.4 and 12.5 years, respectively.^29^ Atypical presentation, that is, non‐amnestic symptom onset was associated with longer median survival in both mutations, despite similar age of onset, particularly for those with *PSEN1* mutation.^29^ Similarly, we found that those with a non‐cognitive presentation in our cohort had longer median survival. It could be that as a psychiatric service, we are inherently biased towards psychiatric symptoms, which may place undue emphasis and interpretation of psychiatric and behaviour changes as part of a dementia syndrome. We were unable to analyse the mortality risks for the different clinical presentations within the major dementia subtypes due to small numbers. Further investigation in genetic forms of YOD, including comparisons with HD, may find modifiers which influence phenotype and outcomes.

We found that females who had YOD have a very high risk of death compared to the general population, and particularly females with FTD and Other Dementias. This finding is consistent with Australian statistics that dementia is a leading cause of death in females.^7^ This could be explained by longer life expectancy in females^30^ and sex differences in phenotype and progression, with females having a more aggressive form of the different types of dementia.^30^, ^31^

Causes of death in HD were unsurprisingly attributed to HD and respiratory disorders.^13^, ^32^ Suicide is also a common cause of death in HD,^13^, ^32^ though we only had one individual with HD who had this as their reported cause of death. The first 3 months of a diagnosis of dementia in someone aged less than 65 years is a particularly vulnerable period, with reports that there is a 3× risk of suicide,^33^ highlighting the need for close follow‐up post‐diagnosis. Amongst our cohort, there were only nine individuals with ‘external’ or ‘unknown’ causes of death, which might be attributed to suicide.

### 4.1 Limitations

Diagnostic investigations and diagnostic criteria evolved over the study period. The study used the contemporaneous diagnosis at the time of initial assessment rather than re‐classifying diagnoses based on current criteria. We did not have pathological confirmation of the dementia diagnoses and while younger people with dementia are known to have diagnostic change, due to follow‐up of our patients, we opt to minimise this change by using the most ‘recent’ dementia diagnosis. We were able to genetically diagnose HD. Retrospective studies are dependent on the information contained within file review, dependent on collateral history for symptom onset type and age of onset, and thus subject to interpretation. Finally, this study was undertaken in a single inpatient setting, where the sample may be more complex or severe, restricting the generalisability of the findings. The sample size of 386 is relatively small, but is the largest Australian study to date. This limitation is potentially a strength, given that we have been able to access a relatively rare group of patients, including with HD, over a long period of time and describe the largest number of YOD patients linked to the Australian NDI.

## 5 CONCLUSIONS

In summary, we studied survival, predictors of mortality and causes of death, in a large cohort of individuals with common types of YOD. We provide survival information which will be of value to patients, families and clinicians for future planning and service provision, and highlight the need for early identification. Supporting these individuals to maintain quality of life during their disease is very important.

## CONFLICT OF INTEREST STATEMENT

The authors declare no conflicts of interest.

## Data Availability

The data that support the findings of this study are available from the corresponding author upon reasonable request.
